# Process evaluation of a mobile phone-based intervention to support post-abortion contraception in Cambodia

**DOI:** 10.1186/s40834-017-0043-8

**Published:** 2017-05-01

**Authors:** Chris Smith, Sokhey Ly, Vannak Uk, Ruby Warnock, Phil Edwards, Caroline Free

**Affiliations:** 1 Department of Population Health, London School of Hygiene and Tropical Medicine, Keppel Street, London, WC1E 7HT UK; 2 Marie Stopes International, Phnom Penh, Cambodia; 3 Department of Obstetrics, Gynecology and Reproductive Sciences, University of California, San Francisco, USA; 4 Room 150, Department of Population Health, London School of Hygiene and Tropical Medicine, Keppel Street, London, WC1E 7HT UK

**Keywords:** Text Message, Medical Abortion, Abortion Service, Effective Contraception, Counsellor Support

## Abstract

**Background:**

The MObile Technology for Improved Family Planning (MOTIF) trial assessed a mobile phone-based intervention comprising voice messages and counsellor support to increase post-abortion contraception at four Marie Stopes International clinics in Cambodia. The aim of this process evaluation was to assess participants’ interaction with the intervention from a service provider perspective.

**Methods:**

(1) We conducted a descriptive analysis to assess participants’ interaction with the intervention. (2) In order to explore how the intervention might work, we assessed associations between interaction with the intervention and contraception use using logistic regression analysis. (3) We undertook a logistic regression analysis to assess associations between baseline socio-demographic factors and *ever* requesting to speak to a counsellor (pressing ‘1’), a variable found to be associated with contraception use.

**Results:**

Amongst 249 women that received six interactive voice messages +/− counsellor support for contraception, around half actively requested to speak to a counsellor (pressed ‘1’) and over 90% spoke to a counsellor at some stage. Women who spoke to the counsellor having requested to (by pressing ‘1’) were more than four times as likely to be using effective contraception at four months compared to women who didn’t request or speak to the counsellor (Odds Ratio 4.39; 95% CI: 1.15-16.71). There was a small, non-statistically significant increase in contraception use amongst women that spoke to the counsellor without requesting a call. Increased parity, a history of >2 previous induced abortions, lower socio-economic status, and medical abortion (after adjusting for age, socio-economic status and residence) were associated with requesting to speak to a counsellor.

**Conclusions:**

The interactive message can identify a subgroup of women in whom counselling will be more effective and appears to be equitable in terms of engaging those most in-need. The intervention could be adapted based on the findings of this study.

## Plain English summary

Our MObile Technology for Improved Family Planning (MOTIF) trial assessed a mobile phone-based intervention comprising six interactive voice messages +/− counsellor support to increase post-abortion contraception at four Marie Stopes International clinics in Cambodia. The aim of this process evaluation was to assess participants’ interaction with the intervention from a service provider perspective to further understand what worked or didn’t work and to inform future implementation. We analysed data collected during the delivery of the intervention. This included women’s response to the messages and number of contacts with the counsellor. We assessed associations between women’s response to the intervention and subsequent contraception use, and associations between women’s background factors and engagement with the intervention.

We found that amongst 249 women that received the intervention, around half actively requested to speak to a counsellor and over 90% spoke to a counsellor at some stage. Women who requested to speak to a counsellor were more likely to be using effective contraception at four months compared to women who didn’t request to speak to a counsellor. Women with living children, a history of >2 previous abortions, of lower socio-economic status, and those who had a medical rather than surgical abortion were more likely to request to speak to a counsellor.

In conclusion, the interactive voice message can identify a subgroup of women in whom counselling will be more effective and appears to be equitable in terms of engaging those most in-need. The intervention could be adapted based on the findings of this study.

## Background

The past decade has seen rapid expansion in delivery of health-care interventions by mobile phone (‘mHealth’) [1]. In the field of contraception, mobile phone-based interventions have been developed to support uptake of methods or reduce discontinuation, for example by providing reminders, or support for clients experiencing side-effects [2]. However, the effects of interventions delivered by mobile phone for improving contraception use have not been reliably established [2].

Our trial, MObile Technology for Improved Family Planning (MOTIF), randomised 500 women seeking elective induced abortion services aged 18 years or older to a personalised mobile phone-based behaviour change intervention or to a control group. The trial results and a detailed description of the intervention and its development are reported elsewhere [3, 4]. In brief, the intervention comprised a series of automated interactive ‘real-time’ voice messages over the three-month post-abortion period in addition to standard care. Women would receive the first message within one week of attending the clinic, and then every two weeks, with a total of six messages. The message was designed to remind women about contraception methods available to them and provide a conduit for additional support [5]. Women could press ‘1’ to request to speak to a counsellor, press ‘2’ if they did not require a call back, or press ‘3’ to opt-out of receiving further messages. Women who pressed ‘1’, or who did not respond, received a phone call from a counsellor. The phone calls provided individualised counselling intending to encourage contraception use by reminding women about available methods and providing support for side-effects. Counsellors could make appointments for implant or intra-uterine device insertions or women could opt to receive additional oral contraceptive pill or injectable reminder messages. The counsellor could discuss contraception with the husband or partner, if the woman requested or women could call in to the service to request to speak with a counsellor.

The intervention increased self-reported use of an effective contraception method at four months post-abortion (135/211 (64 · 0%) vs. 101/220 (45 · 9%) risk ratio (RR) 1 · 39, 95% CI 1 · 17-1 · 66; *p* < 0 · 001), but not after 12 months (84/ (49.7%) vs. 68/ (42.8%) RR 1.16, 95% CI 0.92-1.47; *p* = 0.208). However, self-reported long-acting contraception use was increased at four and 12 months [3].

As a complex intervention delivered by automated voice messages and phone counselling, it is not clear what components of the intervention resulted in behaviour change. Our intervention was based on literature on the determinants of contraceptive use and a similar approach to Lester (2010) who hypothesized that regular structured mobile phone-based support could improve HIV medication adherence [4, 6]. However, for research conducted in ‘real-life’ settings, it cannot be assumed that the delivery of a complex intervention will be exactly as planned in the design stage of a trial. Process evaluation of randomised controlled trials can lead to a greater understanding of what works, and provide meaningful interpretation of the effects on an intervention to inform future implementation [7, 8]. In a subsequent paper we will report findings from interviews conducted with participants that received the intervention. The aim of this study was to assess participants’ interaction with the intervention from a service provider perspective and to consider how the intervention could be improved. Specific objectives were to:

1. Describe the response to voice messages and number of counsellor contacts over the intervention duration
2. Examine associations between interaction with the intervention and subsequent contraception use
3. Assess associations between baseline socio-demographic factors and interaction with the intervention

## Methods

This quantitative study used data collected during the intervention and trial. First, we undertook a descriptive analysis to assess how participants interacted with the intervention. Counsellors delivering the intervention made a record of all mobile phone communications with participants. We report the response to voice messages, the number and type of counsellor contacts and number of women that opted out of the intervention or that the counsellor was unable to contact using descriptive statistics.

Second, in order to explore how the intervention might work, we assessed associations between interaction with the intervention and effective and long-acting contraception use using logistic regression analysis. Using data collected by counsellors delivering the intervention we created variables based on response to the voice messages and whether women spoke to the counsellor or received pill or injection reminder messages. We conducted a pre-specified per-protocol analysis to assess the impact of the intervention among participants who responded to at least one voice message [5]. We estimated odds ratios (OR) with 95% confidence intervals (CI).

Third, we undertook a logistic regression analysis to assess associations between baseline socio-demographic factors and *ever* requesting to speak to a counsellor (pressing ‘1’), a variable that was found to be associated with contraception use in the previous analysis. As the voice message stated *“Press 1 if you would like me to call you back to discuss contraception”* we considered plausible, ‘a priori’ confounders that might be associated with contraception use in Cambodia. Age, socio-economic status, residence, education and number of living children are associated with contraception use in Cambodia and were included in the adjusted analysis [9, 10]. As almost all the women in the trial (>99%) were able to recognise numbers on a phone and spoke Khmer as their mother tongue, we did not consider confounders that might be associated with the ability to understand or respond to a voice message. For categorical variables in the adjusted analysis, we assessed the statistical significance of the crude association, controlled for the effect of the confounding variables using the Likelihood ratio test. Ethical approval for the MOTIF study was obtained from ethics committees at the London School of Hygiene and Tropical Medicine and Marie Stopes International and the Cambodia Human Research ethics committee.

## Results

### Interaction with the intervention

**Fig. 1:**
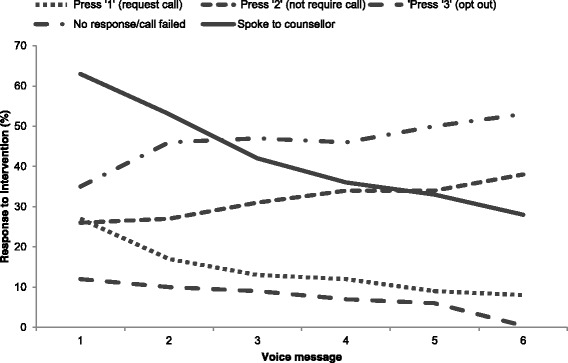
Interaction with intervention over time

Figure 1 shows the response to voice messages over time. The proportion of ‘1’ responses (requesting a call) decreased from 27% at voice message one to 8% by message six. Overall, 49% of clients ever pressed ‘1’ (to request to speak to the counsellor). The proportion of ‘2’ responses (not requiring a call back) increased from 26–38%. The proportion of ‘call failed’ (no response to the voice message) increased from 35–53% at voice message six. The proportion of clients that spoke to a counsellor decreased from 64% at voice message one to 26% at voice message six. In total, there were 210 (15%) ‘1’ responses, 452 (32%) ‘2’ responses, 109 (8%) ‘3’ responses, 657 (46%) ‘no responses’, 613 calls from the counsellor to client (outgoing) and approximately 100 calls from the client to the counsellor (data not systematically recorded). The mean number of outgoing phone calls per client was 2 · 46 (standard deviation 1 · 48). Overall, 92% of participants ever spoke to a counsellor. It is not clear how often the counsellor discussed contraception with the women’s husband or partner, as this information was not systematically recorded.

Twenty per cent (49/249) of participants receiving the intervention opted to receive oral contraceptive or injection reminders at some stage. Of the 24 women that received a reminder message three months after receiving the injectable, 83% (20/24) reported continued use at four-month follow up. Of the 25 women that received monthly oral contraceptive reminders, 68% (17/25) reported continued pill use at four-months.

By voice message six, 15 (6%) clients had opted out. Reported reasons according to the counsellors notes were that they were ‘too busy’ and had ‘no time’, or the ‘phone was answered by someone else’. Six participants (2%) randomised to the intervention did not receive any messages; five due to non-functioning phone number and in one case someone not known to the participant answered the phone.

### Association between interaction with the intervention and contraception use

Table 1 shows associations between interaction with the intervention and effective and long-acting contraception use at four months. The following factors were associated with effective contraception use at four months: Requesting to speak to a counsellor (pressing ‘1’) compared to not pressing ‘1’ after the first voice message (OR 3.37; 95% CI: 1.62-6.98); *ever* requesting to speak to a counsellor (pressing ‘1’) compared to *never* requesting to speak to a counsellor (OR 2.51; 95% CI: 1.41-4.47); speaking to the counsellor having requested to (pressed ‘1’) compared to *never* speaking and requesting to speak to the counsellor (OR 4.39; 95% CI: 1.15-16.71) or speaking to the counsellor having not requested to (OR 1.79; 95% CI 0.47-6.79); received pill or injection reminder compared to not received a pill or injection reminder (OR 4.63; 95% CI: 2.11-10.16). The following factors were associated with long-acting contraception use at four months: Requesting to speak to a counsellor (pressing ‘1’) compared to not pressing ‘1’ after the first voice message (OR 2.05; 95% CI: 1.09-3.88); *ever* compared to *never* requesting to speak to a counsellor (OR 2.88; 95% CI: 1.52-5.45); speaking to the counsellor and *ever* pressing ‘1’ compared to speaking to the counsellor and *never* pressing ‘1’ (OR 2.76; 95% CI: 1.44-5.29).

**Table 1:** Association between interaction with intervention and effective and long-acting contraception use at four months

	Using effective contraception	OR	*p* value	Using long-acting contraception	OR	*p* value
	No/total no. of respondents (%)			No/total no. of respondents (%)		
Per-protocol analysis						
Responded to 1–2 voice messages (r)	33/55 (60%)	1.00		18/55 (33%)	1.00	
Responded to >2 voice messages	89/124 (72%)	1.70 (0.87-3.30)	0.121	40/124 (32%)	0.98 (0.50-1.93)	0.951
Response to voice message 1						
Call failed (r)	40/69 (58%)	1.00		16/69 (23%)	1.00	
1	49/60 (82%)	3.23 (1.44-7.26)	0.005	24/60 (40%)	2.21 (1.03-4.73)	0.041
2	36/58 (62%)	1.19 (0.58-2.42)	0.639	18/58 (31%)	1.49 (0.68-3.28)	0.321
3	10/24 (42%)	0.52 (0.20-1.33)	0.171	3/24 (12%)	0.47 (0.12-1.79)	0.271
Response to voice message 1						
Didn't press '1' (r)	86/151 (57%)	1.00		37/151 (25%)	1.00	
Pressed '1'	49/60 (82%)	3.37 (1.62-6.98)	0.001	24/60 (40%)	2.05 (1.09-3.88)	0.026
Whether participant ever pressed '1'						
Never (r)	53/100 (53%)	1.00		18/100 (18%)	1.00	
1 or more	82/111 (74%)	2.51 (1.41-4.47)	0.002	43/111 (39%)	2.88 (1.52-5.45)	0.001
Spoke to counsellor after voice message '1'						
No (r)	44/74 (59%)	1.00		20/74 (27%)	1.00	
Yes	91/137 (66%)	1.35 (0.75-2.42)	0.315	41/137 (30%)	1.15 (0.61-2.17)	0.658
Whether participant ever spoke to counsellor						
Never (r)	4/11 (36%)	1.00		1/11 (9%)	1.00	
1 or more	131/200 (66%)	3.32 (0.94-11.74)	0.062	60/200 (30%)	4.29 (0.54-34.23)	0.170
Whether pressed '1' if spoke to counsellor						
Spoke to counsellor & never pressed '1' (r)	49/90 (54%)	1.00		17/90 (19%)	1.00	
Spoke to counsellor & ever pressed '1'	82/110 (75%)	2.45 (1.35-4.45)	0.003	43/110 (39%)	2.76 (1.44-5.29)	0.002
Combinations of pressing '1' and speaking to counsellor						
Never pressed '1' & never spoke to counsellor (r)	4/10 (40%)	1.00		1/10 (10%)	1.00	
Never pressed '1' & spoke to counsellor	49/90 (54%)	1.79 (0.47-6.79)	0.390	17/90 (19%)	2.10 (0.25-17.68)	0.496
Pressed '1' & spoke to counsellor	82/110 (75%)	4.39 (1.15-16.71)	0.030	43/110 (39%)	5.78 (0.71-47.22)	0.102
Pressed '1' & never spoke to counsellor	0/1 (0%)	1.00 (.-.)	.	0/1 (0%)	1.00 (.-.)	.
Whether received pill or injection reminders						
Didn’t receive pill or injection reminder (r)	96 (59%)	1.00		60 (37%)	1.00	
Received pill or injection reminder	39 (83%)	4.63 (2.11-10.16)	<0.001	1 (2%)	0.08 (0.01-0.62)	0.015
All participants	135/211 (64%)			61/211 (29%)		

The per-protocol analysis included 179 participants. Participants were categorised as ‘highly protocol adherent’ if they responded (i.e. pressed number ‘1’ or ‘2’) to more than three messages and ‘less protocol adherent’ if they responded to three or fewer messages

### Association between baseline variables and response to the intervention

Table 2 shows the association between socio-demographic baseline variables and response to the intervention. The following socio-demographic factors were associated with ever requesting to speak to the counsellor (pressing ‘1’): Age greater than 25 compared to less than 25 (unadjusted OR 1.78; 95% CI: 1.05-3.02) but not after adjusting for confounding variables; lower compared to higher socio-economic status (unadjusted OR 2.92; 95% CI: 1.23-6.90) which remained statistically significant**;** being married or living together compared to never married or living together (unadjusted OR 4.25; 95% CI: 1.17-15.46) but not in the unadjusted analysis; having one or more compared to no children (unadjusted OR 2.25; 95% CI: 1.30-3.91) which remained statistically significant; having two or more previous abortions as opposed to none (unadjusted OR 3.34; 95% CI: 1.50-7.44) which remained statistically significant; planning to use (unadjusted OR 3.58; 95% CI: 1.09-11.70) or being undecided about PAFP (unadjusted OR 3.71; 95% CI: 1.16-11.82) as opposed to not planning to use PAFP at the time of randomisation but not in the adjusted analysis. Medical compared to surgical abortion became associated with requesting to speak to the counsellor (adjusted OR 1.77; 95% CI: 1.03-3.07) after adjusting for the confounding variables.

**Table 2:** Association between baseline variables and interaction with intervention

	Never pressed '1'	Ever pressed '1'	Crude OR	*p* value	Adjusted OR^a^	*P* value
	*N* = 127	*N* = 122				
Age group						
Age <25 (r)	53 (60%)	35 (40%)	1.00			
Age >25	74 (46%)	87 (54%)	1.78 (1.05-3.02)	0.032	1.06 (0.52-2.19)	0.864
Residence (urban/rural)						
Urban (r)	48 (56%)	37 (44%)	1.00			
Rural	79 (48%)	85 (52%)	1.40 (0.82-2.36)	0.215	1.49 (0.86-2.59)	0.153
Socio-economic status						
Access to motorised transport (r)	119 (54%)	102 (46%)	1.00			
No access to motorised transport	8 (29%)	20 (71%)	2.92 (1.23-6.90)	0.015	3.36 (1.35-8.40)	0.009
Education						
Secondary or above (r)	85 (54%)	71 (46%)	1.00			
None or primary	42 (45%)	51 (55%)	1.45 (0.87-2.44)	0.155	1.02 (0.58-1.80)	0.936
Marital status						
Never married or living together (r)	12 (80%)	3 (20%)	1.00			
Married/living together	112 (48%)	119 (52%)	4.25 (1.17-15.46)	0.028	3.13 (0.76-12.91)	0.091
Divorced/separated	3 (100%)	0 (0%)	1.00 (.-.)	.	1.00 (.-.)	
# living children						
0 (r)	51 (65%)	28 (35%)	1.00			
1 or more	76 (45%)	94 (55%)	2.25 (1.30-3.91)	0.004	2.24 (1.07-4.72)	0.033
# previous abortions						
0 (r)	81 (56%)	63 (44%)	1.00			
1 or more	46 (44%)	59 (56%)	1.65 (0.99-2.74)	0.053	1.68 (0.96-2.94)	0.067
# previous abortions						
0	81 (56%)	63 (44%)	1.00			
1	36 (52%)	33 (48%)	1.18 (0.66-2.10)	0.576	1.26 (0.68-2.33)	0.014
2 or more	10 (28%)	26 (72%)	3.34 (1.50-7.44)	0.003	3.49 (1.45-8.40)	
Previous contraception use						
No (r)	57 (54%)	49 (46%)	1.00			
Yes	70 (49%)	73 (51%)	1.21 (0.73-2.01)	0.452	1.00 (0.57-1.74)	0.997
Contraception decision-making						
Joint decision (r)	70 (51%)	68 (49%)	1.00			
Mainly participant	26 (47%)	29 (53%)	1.15 (0.61-2.15)	0.665	0.89 (0.45-1.76)	0.931
Mainly husband/partner	17 (45%)	21 (55%)	1.27 (0.62-2.62)	0.514	1.06 (0.48-2.35)	
Mobile phone access						
Never shares (r)	65 (52%)	61 (48%)	1.00			
Shares	62 (50%)	61 (50%)	1.05 (0.64-1.72)	0.852	0.99 (0.59-1.68)	0.979
Disclosure of abortion to others						
No (r)	10 (59%)	7 (41%)	1.00			
Yes	52 (49%)	54 (51%)	1.48 (0.53-4.19)	0.457	1.14 (0.36-3.56)	0.825
PAFP intentions						
No (r)	14 (78%)	4 (22%)	1.00			
Undecided	68 (49%)	72 (51%)	3.71 (1.16-11.82)	0.027	2.69 (0.80-9.12)	0.248
Yes	45 (49%)	46 (51%)	3.58 (1.09-11.70)	0.035	2.46 (0.71-8.55)	
Fertility plans						
Have a/another child (r)	82 (53%)	73 (47%)	1.00			
No more/none	34 (50%)	34 (50%)	1.12 (0.63-1.99)	0.690	0.67 (0.34-1.31)	0.388
Undecided	11 (42%)	15 (58%)	1.53 (0.66-3.55)	0.319	1.15 (0.46-2.86)	
Abortion method						
Surgical (r)	80 (54%)	67 (46%)	1.00			
Medical	47 (46%)	55 (54%)	1.40 (0.84-2.32)	0.196	1.85 (1.06-3.22)	0.030
Phone credit						
Always (r)	53 (55%)	44 (45%)	1.00			
Usually	38 (58%)	27 (42%)	0.86 (0.45-1.61)	0.631	0.91 (0.47-1.77)	0.480
Sometimes	36 (41%)	51 (59%)	1.71 (0.95-3.06)	0.073	1.35 (0.73-2.52)	

^a^Adjusted for age, socio-economic status, residence, education and number of living children

## Discussion

### Summary of main results

In summary, amongst 249 women that received six interactive voice messages +/− counsellor support for PAFP, around half actively requested to speak to a counsellor (pressed ‘1’) and over 90% spoke to a counsellor at some stage. Women who spoke to the counsellor having requested to (by pressing ‘1’) were more than four times as likely to be using effective contraception at four months compared to women who didn’t request or speak to the counsellor. There was a small, non-statistically significant increase in contraception use amongst women that spoke to the counsellor *without* requesting a call. Increased parity, a history of >2 previous induced abortions, lower socio-economic status, and medical abortion were associated with requesting to speak to a counsellor (pressing ‘1’) after adjusting for age, socio-economic status and residence, education and number of living children.

### Strengths & limitations

A strength of this study is the use of prospectively collected quantitative data on participant characteristics and response to the intervention which provides some insight into the active components of the intervention. The main limitation of this study is that it only considers a provider perspective and does not consider the views and experiences of users; this will be assessed in a subsequent paper. The main limitation affecting the logistic regression analysis was the relatively small sample size, particularly in some of the subgroups, resulting in lack of statistical power to detect differences. Hence, whilst odds ratios may appear to vary greatly between subgroups, the confidence intervals are wide, and so these trends should be interpreted with caution. It was not possible to adequately evaluate the effect of the pill or injection reminders as they were sent to a relatively small number of women, without a control group, and part of a complex intervention. A further limitation concerns the generalisability of this study and the findings might not be applicable to other settings.

### Interpretation & comparison with existing literature

To our knowledge, this is the first process evaluation of an interactive mobile phone-based intervention for contraception that includes a detailed assessment of provider-participant communication. Other trials of mobile phone-based interventions to improve contraception use have involved unidirectional text messages, and thus provide limited opportunity for comparison [11–14]. However, some comparisons can be made with other studies, in particular the process evaluation of the text message component of Lester’s intervention for antiretroviral medication adherence [15].

### Response to the intervention

Despite sending the messages at the clients preferred time (e.g. morning or evening), the proportion of participants not responding to voice messages (i.e. pressing ‘1’ or ‘2’) was greater than non-responses to interactive text messages reported in trials in Kenya and the USA [6, 14]. The most likely explanation for this is that our ‘real-time’ voice message required an immediate response (there was no voice-mail), whereas clients can respond to a text message at their convenience. The proportion of women actively responding to voice messages decreased over time, similar to decrease in responses observed in previous trials of interactive text message or pager interventions [15–17].

More women requested to speak to a counsellor at voice message one than at message six and the proportion of women not requesting a call back increased over time, which might be expected as issues were resolved. By message six, 15 (6%) clients had opted out mainly because they reported being too busy or shared their phone with someone else, which is common in Cambodia [18]. Six participants (2%) randomised to the intervention did not receive any messages mainly due to non-functioning phone number. A text message trial in the USA reported similar intervention discontinuation (42/480; 9%) and number of participants that never received messages (4/480; 0.9%) [11].

Previous studies have reported greater than 40% discontinuation of short-acting methods by 12 months [19]. In our study, 17% of women that received an injectable reminder message and 32% of women that received a pill reminder message had discontinued at four-months. It is not possible to determine the added value of these additional messages within the whole intervention, as we did not have a comparison group of women using pill or injectable not receiving reminder messages. However, pooled analysis suggests that text message reminders for medication adherence have at best small effects [20]. Elsewhere, a trial in the USA reported that participants receiving text message reminders had a lower mean number of days between scheduled appointment and actual attendance for contraceptive injection for the first, but not subsequent appointments [14].

Assessing associations between interaction with the intervention and contraception use provided further insights regarding possible active components of the intervention. Compared to women that never requested a call (pressed ‘1’) or spoke to the counsellor, women who pressed ‘1’ and spoke to the counsellor were over four times more likely to be using effective contraception at four months. In contrast, there was a lesser, non-statistically significant increase in contraception use amongst women that spoke to the counsellor *without* requesting a call. We did not find evidence that contraception or fertility intentions at the time of seeking abortion services were associated with requesting to speak to a counsellor, after adjusting for the confounding variables. The finding that phoning women who requested a call is associated with subsequent use of effective contraception suggests that the interactive message can identify a subgroup of women in whom counselling will be more effective.

### Public health implications

Our finding that women were more likely to request a call back from a counsellor (pressing ‘1’) if they were of lower socio-economic status or increased parity suggests that the intervention is equitable in terms of engaging those most in-need and could have public health benefits at scale. Older women and those with increased parity are at greater than average obstetric risk from subsequent unintended pregnancies [21]. Poor women are most likely to experience complications related to unsafe abortion from subsequent unintended pregnancies [22].

Age was associated with pressing ‘1’ in the unadjusted analysis was because older women were poorer, less educated, had more children or previous abortions and more likely to live in rural areas. In contrast to other studies, we didn’t find that residence was associated with response to the voice message [15]. However, it is possible that lack of access to motorised transport (proxy for socio-economic status) was a barrier to accessing face-to-face health services.

Women with one or more child might have requested to speak to a counsellor due to increased motivation to prevent another unintended pregnancy. Engagement by women who have had several abortions could reflect problems with contraception in the past or post-abortion health concerns. Women opting for medical abortion might be more likely to request to speak to a counsellor for support regarding managing their symptoms at home [23]. Although it is safe to use a full-range of contraceptive methods apart from intra-uterine device on the day of medical (misoprostol) treatment, [24] some women might want to postpone decisions about contraception use until the abortion is complete.

### Implications for practice/research

First, given that women were more likely to subsequently use contraception if they requested to speak to a counsellor (i.e. pressed ‘1’), the intervention could be further refined so that counsellors only phone women that request to speak to a counsellor. As the cost of counselling is likely to be the main limitation to scaling up the intervention, this change would reduce costs but any effect of the intervention amongst women that speak to the counsellor *without* requesting a call would be lost. Second, in settings where smartphones use is high, the intervention could be adapted whereby the voice message is sent via an instant messaging application and listened to at the woman’s convenience, and can be listened to several times. This might increase the response rate to the messages. Use of such applications provides additional opportunities to add other low-literacy content such as stickers/cartoons and future research could evaluate such interventions.

## Conclusions

The interactive message can identify a subgroup of women in whom counselling will be more effective and appears to be equitable in terms of engaging those most in-need. The intervention could be adapted based on the findings of this study.
